# Clinical Implications of Growth Hormone Deficiency for Oral Health in Children: A Systematic Review

**DOI:** 10.3390/jcm10163733

**Published:** 2021-08-22

**Authors:** Natalia Torlińska-Walkowiak, Katarzyna Anna Majewska, Andrzej Kędzia, Justyna Opydo-Szymaczek

**Affiliations:** 1 Department of Pediatric Dentistry, Poznan University of Medical Sciences, Bukowska 70, 60-812 Poznan, Poland; jopydo@ump.edu.pl; 2 Department of Clinical Auxology and Pediatric Nursing, Poznan University of Medical Sciences, Szpitalna 27/33, 60-572 Poznan, Poland; katarzynamajewska@ump.edu.pl (K.A.M.); akedzia@ump.edu.pl (A.K.)

**Keywords:** growth hormone deficiency, children, caries, dental maturation, craniofacial growth/morphology, tooth wear, enamel defects

## Abstract

Growth hormone (GH) is involved in the regulation of the postnatal dental and skeletal growth, but its effects on oral health have not been clearly defined. This paper aims to provide a review of current clinical knowledge of dental caries, tooth wear, developmental enamel defects, craniofacial growth and morphology, dental maturation, and tooth eruption in growth hormone deficient (GHD) children. A systematic review was carried out using Scopus, MEDLINE-EbscoHost and Web of Science from 2000 to May 2021. PRISMA guidelines for reporting systematic reviews were followed. All the selected studies involved groups under eighteen years of age, covering a total of 465 GHD patients. The studies that were selected provide reliable evidence for delayed dental maturity and orthodontic disturbances in GHD patients. Data on dental hard tissues pathology are scarce and are limited to occurrences of dental caries. GHD children showed abnormal craniofacial morphology with reduced mandibular dimensions, with a resulting tendency towards Angle’s Class II occlusion, which affected up to 31% of patients. Dental age has been shown to be delayed in GHD patients by about 1 to 2 years. Moreover, the risk of dental caries in children with GHD decreases with increasing levels of vitamin D. Hence, further studies would be valuable for evaluating the risk of various oral health problems and to organize targeted dental care for this vulnerable group.

## 1. Introduction

Tooth development and eruption, as essential parts of general development, are examples of processes that can be easily disturbed. It is well known that the development of the alveolar bone surrounding the tooth germs is closely coordinated with tooth morphogenesis. At the same time, eruption also depends on precisely regulated bone remodeling [1,2].

Growth hormone (GH) is a critical regulator of the growth process in children. It is secreted by the pituitary gland, mainly during physiological night sleep. GH exerts its function mainly by promoting insulin-like growth factor I (IGF-I) secretion, acting within the GH-IGF-I signaling axis [3,4].

Despite numerous studies and the seemingly clear effect of GH on dental development, this process is still not well understood [5]. Research indicates that growth hormone action is associated with tooth maturation and eruption [6,7]. GH is able to induce proliferation of epithelial stem cells in molar buds, along with preameloblast differentiation and enamel formation [8,9]. GH and IGF-I induce the production of morphogenetic proteins 2 and 4 (BMP-2, BMP-4) and of the transforming growth factor-beta superfamily, affecting odontoblast differentiation and osteodentin and tubular dentine formation [8,10,11]. Furthermore, it has been shown that GH affects cellular cementum in mice [12,13]. GH, GH receptor, and IGF binding proteins are detected during particular phases of tooth development, at the bud stage, cup stage and bell stage, affecting the tooth shape and size [8,14].

Both, cell sensitivity to GH and the site of GH action are closely coordinated and affect the odontogenesis. When the new matrix begins to form, GH receptors are expressed in tooth tissues and mediate local growth responses. However, cementocytes and mature odontoblasts in later stages of tooth development do not display expression, which suggests that they become insensitive [3].

Recent studies have shown that IGF-I and its receptor are expressed in both dental epithelial and mesenchymal tissues of tooth germs, increasing the size of bioengineered tooth germs. In vitro IGF-I signaling promotes cell proliferation, differentiation, and matrix secretion in mouse tooth germs, and so it can be hypothesized that IGF-I regulates tooth morphogenesis [4,15]. 

Oyanagi et al. [15] showed that the combination of IGF-I and BMP2 promotes odontoblast differentiation and the expression of the ameloblastin enamel matrix gene (*Ambn*) in mice. Their research demonstrated that the expression of *Ambn* is directly enhanced by IGF-I in dental epithelial cells, so *Ambn* may play an essential role in normal ameloblast differentiation. It is interesting that at least a hundred genes have been recognized as being expressed during different stages of amelogenesis; this total includes genes that codify sex hormone receptors and growth hormone receptors [16]. Estrogen plays an essential role during tooth formation by influencing the process of enamel and dentin mineralization. An animal model of estrogen deficiency showed a significant reduction in enamel microhardness [16]. The results reported by Arid et al. [16] in humans have demonstrated that genetic polymorphism in estrogen receptors (rs12154178) and in GH receptors (rs1509460) is associated with alterations in ameloblast function and developmental defects of enamel (DDE). It must be remembered that disturbances in enamel formation are associated with poor esthetics and the higher susceptibility of teeth to harmful local factors. Developmental enamel defects increase the risk of dental caries and noncarious tooth surface loss, which affect long-term dental health [17,18]. The posteruptive onset of the caries process takes part for as long as three years after permanent tooth eruption [19]. Caries in primary teeth shows a rapid course, due to the specific morphological structure and a lower degree of mineralization. Effective prevention and treatment, especially in the case of primary dentition, can considerably extend the time that a tooth remains in the oral cavity, thus preventing the consequences of its premature loss. In patients with growth hormone deficiencies, missing teeth and tooth-bone discrepancies become risk factors for masticatory organ disorders.

Isolated growth hormone deficiency (IGHD) may be caused by various genetic factors or by structural changes in the hypothalamus or pituitary, but the most commonly diagnosed form of the disease is idiopathic, with an unknown cause [20,21,22,23]. The main symptoms of IGHD include short stature and poor growth velocity [22,24,25]. Moreover, the length and depth of the face are generally inappropriately small for the child’s age. Some studies have reported that mandibular total length is reduced, primarily as a result of small ramus height. In addition, the maxilla, although reduced and often retrognathic, is affected less than the mandible [2]. Disturbances such as agenesis and crown anomalies are also observed, aside from enamel and dentin malformation [8,26]. Children who received long-term GH therapy (for over two years) show increased growth of the craniofacial skeleton, and especially of the maxilla and mandibular ramus. These findings suggest that GH accelerates craniofacial development, which improves occlusion and facial profile [2]. 

Data from the literature confirm that dental age is delayed with respect to chronological age by up to two years in growth hormone-deficient (GHD) children [27]. It has been reported that tooth eruption, defined as the mucosal penetration of any visible part of a tooth into the oral cavity, is delayed in both primary and permanent dentition [27,28]. The relationship between the maturation of the skeletal system and the formation of permanent dentition has been confirmed in several studies [29,30]. There is a relationship between mineralization of the canine and the MP3 stage—one of the stages of ossification of the middle phalanx of the third finger in the development of bones of the wrist [29]. Moreover, the relationship between dental age and maturity in the cervical vertebrae is also determined. A significant association has been confirmed between cervical vertebral maturation (CMV) classification and the development of canine and second premolar teeth [31]. At the same time, it should be remembered that the time for emergence of permanent premolars and canines can be modified by the activity of caries in primary teeth. Several studies have concluded that early extraction of second primary molar or caries in primary molars can accelerate the clinical eruption of permanent second molars [32]. 

The study aimed to conduct a systematic review of knowledge of oral health relating to craniofacial development, occlusion, dental age, dental caries, enamel defects, and tooth wear in children with GHD. 

## 2. Materials and Methods

### 2.1. Inclusion and Exclusion Criteria

The literature search was based on a previously prepared protocol that defined inclusion and exclusion criteria, a search strategy, and the data analysis [33]. The systematic review protocol was registered with PROSPERO, registration number: CRD42021250229 [34].

This systematic review of the available literature on the condition of the teeth of pediatric GHD patients in terms of dental caries, tooth wear, enamel defects, and dental maturity and tooth eruption, craniofacial growth and morphology, and malocclusion was carried out in accordance with Cochrane Collaboration guidelines [33]. 

**Figure 1:**
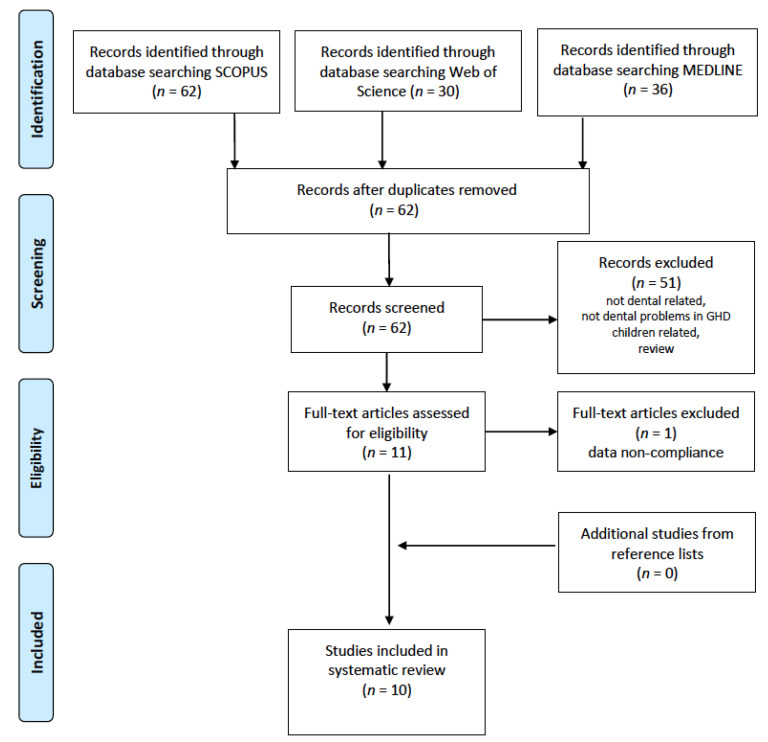
PRISMA flowchart of the study selection process.

Human clinical trials and observational studies related to dental caries, tooth wear, enamel defects, tooth eruption and dental maturity, malocclusion, and craniofacial growth and morphology in pediatric GHD patients were included. The exclusion criteria: (1) nonhuman studies and in vitro studies; (2) subjects with syndromic short stature, idiopathic short stature (ISS) or neoplasia; (3) case reports and reviews; (4) editorials, commentaries, books, and letters to editors; and (5) articles without available full text. The search strategy was based on the Preferred Reporting Items for Systematic Reviews and Meta-analyses (PRISMA) guidelines [35] (Figure 1).

### 2.2. Search Strategy

We searched Scopus (2000 to May 2021), MEDLINE-EbscoHost (2000 to May 2021), and Web of Science (2000 to May 2021). As the aim was to present the most recent summary of evidence, the analysis covered publications from the last 20 years, from January 2000 to May 2021, in English or Polish. The present systematic review has been designed to answer the question “Are children diagnosed with GHD, when compared to heathy or GH treated children, more often affected by oral health problems?” formulated according to PICO (“Population”, “Intervention”, “Comparison”, “Outcome”) [36]. The search strategy was carried out using MeSH (Medical Subject Headings) synonyms and Boolean logical operators. The following terms were used in search engines: “growth hormone deficiency” AND (“enamel” OR “tooth wear” OR “caries”); “growth hormone deficiency” AND “dental maturity”; “growth hormone deficiency” AND “craniofacial morphology”; “growth hormone deficiency” AND “craniofacial growth”; “growth hormone deficiency” AND “malocclusion”. The first search was made on 5 January 2021 and the final one on 2 May 2021. 

Studies were selected independently by two review authors (N.T.-W. and K.A.M.) Eligibility was determined by discussion where there were discrepancies. We searched manually for additional studies by cross-checking the reference lists of all the included studies. Duplicate publications were removed. Two of the review authors (N.T.-W. and K.A.M.) independently screened titles and abstracts of all the identified articles so that each record was checked twice. If a title and abstract met the inclusion criteria, the full texts of all potentially relevant articles were retrieved. Full-text articles were reviewed in detail and independently for eligibility criteria by two review authors. 

### 2.3. Data Extraction and Management

Two review authors (N.T.-W. and K.A.M.) independently extracted all the relevant data from the eligible studies and recorded it on a specifically designed form. Any discrepancies were resolved by discussion. If no agreement could be reached, arbitration was carried out by a third review author (J.O.-S.) Data extraction included the following: name of authors, country of patients, year of publication, study group, control group, study design, prevalence of dental caries, tooth wear, tooth eruption and dental maturity, enamel defects, malocclusion, and craniofacial growth and morphology in pediatric GHD patients.

To evaluate the risk of bias, reviewers independently (N.T.-W., K.A.M) evaluated the methodological quality of the studies using the adopted version of Newcastle-Ottawa Quality Assessment Scale according to a star-based system [37,38]. Any discrepancies were resolved by the third author (J.O.-S.) Each study was judged on three categories: the selection of the study groups, the comparability of the study groups, and the ascertainment of the outcome. A study could be awarded a maximum of two stars for the comparability category, the ascertainment of the factor and the assessment of outcomes items and a maximum of one star for each other numbered item within the selection and outcome categories. 

The following criteria of reliability were used: ≥7 stars represented a low risk of bias (good quality study), 5–6 stars a medium risk of bias (fair quality study), and ≤4 stars a high risk of bias (poor quality study).

## 3. Results

After duplicates were manually eliminated, our systematic search of the three medical databases yielded a total of 62 publications meeting the search criteria. An initial selection of these was made using their titles and abstracts.

A total of fifty one articles were excluded because they focused on topics other than dental status, they did not study pediatric GHD patients, or they were reviews. Based on the full text, ten publications were qualified for further analysis; two of these concerned the mineralized tissues of the tooth–dental caries [39,40], two dealt with dental maturity [28,41], two with malocclusion [28,42], and six with craniofacial growth or morphology. One paper was excluded because of inconsistent data [2,28,43,44,45,46] (Figure 1).

Two articles worked with the same group of patients [39,40]; for this reason, some results were considered only once.

The publications describe patients living in North America, Asia, and Europe [2,28,39,40,41,42,43,44,45,46] (Table 1). All the studies involved patients from 5 to 18 years of age, covering a total of 465 GHD and 51 ISS (idiopathic short stature) individuals. Patients with ISS, as described by Choi et al. [45], Hodge et al. [42] and Kim et al. [43], were not considered in our analysis. Two studies (by Segal et al. [46] and Hodge et al. [42]) provided no information on the sex of the patients. One study by Kjellberg et al. [28] dealt only with male patients. Except for the articles of Segal et al. [44], Hodge et al. [42], Choi et al. [45], and Kim et al. [43], the studies all described only GHD and untreated GHD patients. In Segal et al. [46], eleven patients had multiple pituitary hormone deficiencies. The control groups in the studies included healthy children [41,43,45] or relatives [46], reference materials [2,28,44], medical records [42], and study groups divided into subgroups [39,40,45,46]. In five articles the researchers were dentists or dental hygienists [28,39,40,43,45]. Two papers [39,40] included analysis of vitamin D levels. Other vitamin and mineral deficiencies were not assessed.

### 3.1. Hard Mineralized Tissue Pathology

Dental caries were examined in two publications presenting the same patient cohort [39,40] (Table 2). The dental examinations were carried out in line with World Health Organization (WHO) criteria for epidemiological studies. The severity of dental caries was assessed using the DMFT index, which identifies those teeth (T) which have cavities (D); are missing (M); or have been filled (F) as a result of caries. A statistically significant effect of vitamin D3 concentration on the DMFT index and its component DT was found among children from rural areas, where an increase in vitamin D3 concentration by ten units resulted in a decrease in the value of DMFT by 0.82 and a decrease in the value of DT component by 0.66. The percentage of these children with active caries was higher than in urban areas, but not statistically significantly [39]. A positive and statistically significant correlation between the duration of GH therapy and DMFT index was, however, observed in patients from urban areas [40]. There was no healthy control group in this study.

### 3.2. Dental Maturity and Malocclusion

Two of the papers we considered describe the prevalence of malocclusion in GHD children [28,42] (Table 3). Both used the relations of first permanent molars (Angle’s classification) to detect deviations from Angle’s Class I occlusion, where the mesiobuccal cusp of maxillary first molar occludes in the buccal groove of the mandibular first molar. In the study of Kjellberg et al. [28], 29% of the boys in the study group showed Angle’s Class II malocclusion, while the remainder were Angle’s Class I. Dental crowding of at least 2 mm was recorded in 44% of patients. A large overjet (>6 mm) was seen in 14%, and a large overbite (>5 mm) in 5%.

Hodge et al. [42] observed Angle’s Class II in 31% and Angle’s Class III in 6% of patients. Increased overjet and deep overbite were each found in up to 37% of subjects, which is a significantly greater prevalence than in Kjellberg et al. [28] However, these discrepancies are probably due to differences in methodology and definitions. In the study of Hodge et al. [42], an overjet greater than 2 mm and an overbite greater than 3 mm were considered abnormal, while Kjellberg et al. [28] noted only more extreme abnormalities. Unlike Hodge et al. [42], Kjellberg et al. [28] used radiographs and plaster models to record relations between the jaws.

Dental maturity was evaluated in two studies by Kjellberg et al. [28] and Partyka et al. [41] Each investigator used a different method: the method of Demirjian was employed by Kjellberg and the method of Matiegka and Lukasova by Partyka; both of which were validated. Kjellberg et al. [28] defined dental maturity on the basis of tooth formation recorded on orthopantomograms. The sum of scores for each individual was converted into a dental age in accordance with the instructions given by Demirjian. The method of Matiegka and Lukasova established dental age by identifying the most recently erupted full group of teeth, including incomplete groups. From Matiegka’s table for boys and Lukasova’s for girls, age corresponding with the number of teeth can be found, giving a result for a specific patient [41]. Both studies showed statistically significant differences between birth age and dental age between the GHD and non-GHD patients and control groups [28], and between birth age and dental age in patients starting treatment [41]. In Kjellberg et al. [28], dental maturity was delayed about one year in both the non-GHD and GHD boys. Partyka et al. [41] reported a delay of 18.82 and 2.70 months (for the group starting treatment and that undergoing treatment, respectively).

### 3.3. Craniofacial Growth/Morphology

Six articles on craniofacial growth and morphology were included in the systematic review [2,28,43,44,45,46] (Table 4). Five publications used lateral cephalograms to measure the results [2,28,43,44,45]. The number of landmarks and the linear and angular measurements differ between the studies, and three articles mentioned the methods used: Kjellberg et al. [28] used the Bjork method, Choi et al. [45] used Pancherz’s method, and Segal et al. [46] used the triangulation methods developed by Bookstein. Anterior cranial base length was found to be significantly reduced by Preda et al. [44], Kim et al. [43], and Choi et al. [45], while the posterior cranial base length was shorter in Kjellberg et al. [28], Preda et al. [44], Choi et al. [45], and Kim et al. [43] Total cranial base length was significantly less in Preda et al. [44] and Kim et al. [43] Lower anterior facial height was significantly smaller among boys and girls prior to and during treatment in Funatsu et al. [2] Segal et al. [46] also found smaller vertical proportions, suggesting a deficiency in the lower face. Both mandibular ramus height and corpus length were shorter in boys prior to treatment in Choi et al. [45] Smaller mandibular corpus lengths were noted by Preda et al. [44] among boys and girls, and prior to and during treatment in Funatsu et al. [2], while mandibular ramus lengths were shorted in untreated boys in Kim et al. [43] The measured angles referred to the mandible’s and maxilla’s retroposition [28,43,44]. Significant differences between the studied group were also apparent in the angle between the maxillary and mandibular planes, which was larger than normal [43,44,45]. 

### 3.4. Quality Assessment and Risk of Bias

All studies were classified in accordance with the Cochrane collaboration guidelines [33]. A control group was used in all publications, although its size and structure was not always consistent. While only three groups were gender-paired [2,28,45], most of them were similar to the study group in terms of age [2,28,39,40,42,43,44].

The control group in the study by Kjellberg et al. [28], Segal et al. [46], Hodge et al. [42], and Preda et al. [44] consisted of children from previous studies. None of the publications described the blindness of examiners. Intrarater and interrater reliability were calculated in Kjellberg et al. [28] and Choi et al. [45] All papers performed statistical analysis, although not all aspects were statistically analyzed in one of the studies [28]. 

The publications on malocclusion [28,29] were found to be at medium risk of bias. Two articles on dental caries describe the same cohort of children and present similar conclusions [39,40]. One of them was classified as a good quality study, since it additionally included a sample size calculation [40]. One publication on craniofacial characteristics [44] was at medium risk, and five [2,28,43,45,46] were at low risk. One publication on dental maturity [41] was at medium, one at low risk [28] (Table 5).

## 4. Discussion

Through their influence on bone metabolism, GH and IGF-I are major regulators of postnatal growth and development. GH acts directly on tissues by means of GH receptors, or indirectly by the production of insulin-like growth factor I. Metabolic agents and the growth hormone/insulin-like growth factor-I axis have a strong influence on the metabolism of oral tissues, particularly during the period of growth [8]. 

There is very little in the literature on oral cavity status in patients with GHD. Our systematic review has shown that some dental topics have not yet been discussed. There has been little to evaluate dental conditions like tooth wear and enamel defects, although we can assume that the effect of growth hormone on the dentition and facial bones is complex [3,8]. Publications on dental status and craniofacial growth in children with GHD are somewhat confined, and their results are not always concordant [6].

Some researchers have suggested a relationship between tooth wear and craniofacial morphology, as well as a correlation between tooth wear and malocclusion [47,48]. Tooth wear is defined as the mechanical or chemical removal of dental hard tissues, resulting in reduced tooth structure. The prevalence and severity of tooth wear in contemporary populations is on the increase, particularly in younger patients [49]. Patients with significant tooth wear have been described as having a characteristic craniofacial morphology. Cephalometric analysis has reported a reduction in lower anterior facial height, a more horizontal mandibular plane angle, a more acute gonial angle, and a greater posterior facial height [47]. It would be useful to examine these features in GHD children.

The size, growth, and osseous maturity of the jaw also play a role in the process of tooth eruption. A strong correlation has been shown between eruption time and dental maturity. The teeth typically erupt when they have reached a 2/3 root length [50], but individual correlation between chronological age and eruption time is inconsistent [50,51]. Research has demonstrated that, in GHD patients, dental age (maturity) is significantly delayed [28,41]. This is consistent with the results of Cantu et al. [27], which indicated a mean delay in dental age of close to one year. Furthermore, they observed no significant effect of GH treatment on dental maturation. The lack of a subsequent therapeutic response would indicate that dental age is less affected by GH than craniofacial growth.

It thus appears that the increasing maturity of teeth and eruption requires further investigation. It can be assumed that the tooth maturation process and the eruptive movements of the tooth after crown formation are endocrinologically controlled [50]. 

The studies involved in our systematic review report that not only the height of GHD children, but also their craniofacial morphology and growth, are affected [43]. These studies support the previously demonstrated idea that the linear growth of the body is strongly correlated with jaw growth [49], and that the growth of craniofacial skeletal structures is poor in periods of slow longitudinal growth [2,28,43,44,45,46]. 

Several linear craniofacial measurements have been found to be shortened in GHD patients, particularly the mandible and the cranial base [2,28,43,44,45,46]. In children with GH deficiency, it is the mandible that is small, especially the ramus length [2,43]. The most pronounced facial growth retardation is found for posterior face height [43].

Due to mandibular growth retardation, the mandible can be rotated backwards, and the dental-alveolar compensatory mechanism can be activated vertically in the anterior region, in order to maintain incisal contact for as long as possible [6]. It has been observed that males with GHD prior to treatment had a tendency to exhibit skeletal Class II [45]. However, Angle’s Class II malocclusion was not as prevalent as expected given the retrognathic mandibular positions and reduced mandibular dimensions seen in many of the boys. This may be explained by the ability of the occlusion to adapt to slow changes during growth [28].

GH therapy induces the most pronounced catch-up growth within the first one to two years. Funatsu et al. [2] stated that GH therapy was started at a younger age in those in the long-term therapy group than in those in the untreated or short-term therapy groups. It was postulated that the GHD in the long-term therapy group was more severe than in the others. This is in agreement with the conclusion of Cantu et al. [27], which postulated that catch-up may depend not just on growth potential, but also on accumulated growth deficits at the beginning of growth hormone replacement therapy [52].

Dental caries have still not been extensively studied in GH-deficient children. Caries resistance was first suggested by Nikiforuk et al. [53], who concluded that the etiology of this condition most probably lay in the increased maturation time of enamel tissue before the eruption and the reduced exposure to environmental factors. The more recent study by Schroth et al. [54] reports that caries-free children were twice as likely to have optimal 25 (OH)D concentrations (>75 nmol/L), and those with caries presented deficient levels (<35 nmol/L). This was confirmed by Wójcik et al. [39,40], who related the lower prevalence of caries in GHD children the higher 25 (OH)D level. It was concluded in those studies that an increase in vitamin D3 concentration by ten units decreased DMFT by 0.82 and DT by 0.66. The relatively poor range of data on the level of caries in GHD patients suggests the need for further observations.

The occurrence of dental caries and tooth wear should be studied further in cases where the primary teeth remain longer in the oral cavity. After all, we know that the condition of the mineralized teeth tissues in both generally healthy patients and in those with GHD transfers across to the condition of the stomatognathic system. The healthier the masticatory organ, the better the condition of pediatric patients entering adulthood.

Birth age does not reflect fully the physiological development of a child. In order to closely evaluate the process of growth, it is necessary to use other criteria, such as dental age and skeletal age. These parameters are essential for dental providers to provide diagnoses and to plan therapy. In clinical practice, evaluation of both dental and skeletal age would be valuable in all children undergoing dental treatment, especially in those with GHD.

This study has several limitations. The first is the small number of GHD children examined in some of the studies, which makes the results difficult to compare. The studied groups differed in the number of participants, and sometimes the results of a much larger number of children were analyzed in the control group [28,42,44,46]. Although children with various medical conditions were excluded, the control groups did not only contain healthy children, and the comparison was made between the children both at the beginning of treatment and during treatment. The lack of information on the conditions of the dental examinations, especially regarding the qualifications of the examiners, makes the studies prone to bias. Our study search strategy was limited to English and Polish language papers from the last 20 years; this approach may have resulted in the omission of some reports, but it would be difficult to relate the results of studies conducted over twenty years ago to current conditions.

## 5. Conclusions

The available studies indicate that children with GHD showed abnormal craniofacial morphology with reduced mandibular dimensions, with a resulting tendency to Angle’s Class II occlusion, which affected up to 31% of the patients. Dental age has been shown to be delayed in GHD patients by about 1 to 2 years. Moreover, the risk of dental caries in children with GHD decreases with increasing levels of vitamin D. The data are scarce and further studies would be valuable in evaluating the risk of various oral health problems and in organizing targeted dental care for this vulnerable group.

To gain more of an insight into the effects of this disease and its treatment on oral health and craniofacial structures, data need to be collected both before and after GH administration. Such longitudinal studies could help us to understand the complex endocrine mechanisms regulating the stomatognathic system’s development and functions, in order to provide the optimal treatment of GHD-related disturbances.

## Figures and Tables

**Table 1:** Studies included in the systematic review.

Reference	Country	GHD Group Patients and Age	Control Group Patients and Age	Number/Qualification of Examiner(s)	Statistical Methods
Kjellberg et al. (2000) [28]	Sweden	48 boys; 20 GHD 12.1 ± 1.7 years, range 9.1–14.8; 28 non-GHD 11.6 ± 1.7 years, range 7.3–15.0	Reference materials; sagittal cephalograms -109 healthy controls, 12.0 years; PA-cephalograms -588 Australian schoolchildren 6–15 years; 67 boys,12–13 years; occlusion-686 boys, 12 years; dental maturity-453 children; Tooth eruption-122 randomly selected boys; 2–18 years	2/dentist-unknown	Unpaired *t*-test, paired *t*-test Areas in two tails of the standard normal curve Statview v.4.5
Segal et al. (2004) [46]	USA	52 GHD subjects: 41 isolated GHD, 11 multiple pituitary HD; 12.8 ± 4.3 years	41 healthy first-degree relatives; 12 untreated subjects with GHD, 9.2 ± 3.8 years; 2953 healthy controls, 0–18 years (literature reference data)	1/unknown	*t*-test; non-parametric tests; post-hoc comparison linear regression
Funatsu et al. (2006) [2]	Japan	57; 33 boys, 24 girls; untreated group 9 boys and 8 girls, 10.9 ± 3.05 years; short-term therapy group 10 boys and 7 girls,11.4 ± 2.77 years; the long-term therapy group 14 boys and 9 girls, 12.4 ± 2.93 years	Individuals of the same sex and similar chronological age (literature reference data)	Unknown	*t*-test, multiple comparison test (Fisher PLSD); one-way analysis of variance
Hodge et al. (2015) [42]	USA	16; 12 GHD, 3 IGFD + 1 ISS; 12.9 years, range 5–14.11 years	The sample of the U.S. population examined during the National Health and Nutrition Survey (NHANES) (medial records)	Unknown	*t*-test
Choi et al. (2017) [45]	South Korea	36; 10 boys and 8 girls in both group; 18 GHD 18 ISS, 11.3 ± 1.8 years	The same 18 GHD 11.3 ± 1.8 years; Data of 18 healthy children selected from elementary schools in Daegu-data selected to fit short stature children	1/dentist	Shapiro–Wilk test, Kruskal–Wallis test, Mann–Witney U test, Spearman’s rank correlation coefficients IBM SPSS 21.0
Wójcik et al. (2018) [39] Wójcik et al. (2019) [40]	Poland	121; 92 boys,29 girls; 13.73 ± 2.40 years, range 6–18 years	The study group divided by residence into 2 comparative groups- from urban and rural areas: 56 rural areas 65 urban areas	unknown/dentist	Kruskal–Wallis test, Mann–Witney U test, Spearman’s rank correlation coefficients; *t*-test; Pearson’s linear correlation coefficient; Pearson’s Chi2 test Statistica 10 software package
Partyka et al. (2018) [41]	Poland	110; 27 males, 83 females; 13 ± 2.6 years	41 generally healthy children hospitalized in the Department of Pediatric Otolaryngology; 15 males, 25 females; 11.5 ± 2.5 years	unknown	Statistica 10 software package
Preda et al. (2019) [44]	Romania	13 isolated GH deficient; 9 boys, 4 girls; range 9–13 years	Data from the literature regarding the age of the subjects and the pathology	1/unknown	Shapiro–Wilk and Anderson–Darling tests; *t*-test; analysis of variance (ANOVA) Statistical Package for Social Sciences (SPSS)
Kim et al. (2021) [43]	Korea	63; 31 growth hormone deficient (SS-HD); 16 male, 15 female; 10.35 ± 1.84 years; 32 idiopatic short stature (SS-I), 10.31 ± 1.82 years	32 NC (normal children), 17 males and 15 females, who had visited the dental clinic in Daegu, 10.31 ± 1.82 years	2/dentist and hygienist	Multivariate analysis of variance (ANOVA) IBM Statistical Package for Social Sciences (SPSS)

**Table 2:** Dental caries experience—data abstracted from studies included in systematic review.

Reference	GHD Patients Rural Areas	Control Group—GHD Patients Urban Areas	Conclusions
Wójcik et al. (2018) [39] Wójcik et al. (2019) [40]	DMFT = 4.36 ± 2.98 rural DMFT from 0 to 12	DMFT = 3.82 ± 3.76 urban DMFT from 0 to 16	The percentage of rural patients with active caries is higher than of urban patients, but not significantly different (*p* = 0.11). No significant impact of vitamin D3 concentration on DMFT in urban areas. The statistically significant impact of vitamin D3 concentration on DMFT in rural areas (*p* < 0.05). Significant impact of vitamin D3 concentration on the value of DT component (*p* = 0.023).
	Vitamin D3 concentration lower than 10 ng/mL DMFT = 7.67 ± 2.08 DT = 4.67 ± 3.73 Vitamin D3 concentration lower than 20 ng/mL DMFT = 5.55 ± 2.56 DT = 3.78 ± 2.68 Vitamin D3 concentration lower than 30 ng/mL DMFT = 4.08 ± 2.26 DT = 1.38 ± 1.27 Vitamin D3 concentration higher than 30 ng/mL DMFT = 3.70 ± 3.05 DT = 1.93 ± 1.66	Vitamin D3 concentration lower than 10 ng/mL DMFT = 1.00 ± 0.33 DT = 0.50 ± 0.71 Vitamin D3 concentration lower than 20 ng/mL DMFT = 4.25 ± 3.35 DT = 1.31 ± 1.94 Vitamin D3 concentration lower than 30 ng/mL DMFT = 3.93 ± 1.96 DT = 1.85 ± 2.07 Vitamin D3 concentration higher than 30 ng/mL DMFT = 2.95 ± 2.21 DT = 0.79 ± 0.88	
	Increase in vitamin D3 concentration by 10 units decrease in the value of DMFT by 0.82, of DT component by 0.66.		
Wójcik et al. (2019) [40]	DMFT index vs. duration of GH therapy—no correlation	DMFT index vs. duration of GH therapy—formula: DMFT = 1.49 + 0.07 *duration of therapy	The statistical significant correlation between the duration of the GH therapy and the DMFT index among patients from urban areas (test for Pearson’s correlation coefficient *r* = 0.33, *t* = 2.79; *p* = 0.007) No statistically significant correlation between the duration of the GH therapy and the DMFT index among patients from rural areas (test for Pearson’s correlation coefficient *r* = 0.11, *t* = 0.87; *p* = 0.38)

**Table 3:** Malocclusion and dental maturity—data abstracted from studies included in the systematic review.

Reference	Methods of Study	GHD Patients	Control Group	Conclusions
Kjellberg et al. (2000) [28]	*Malocclusion* recorded on plaster model Angle’s Class II defined as cusp to cusp relation or full Class II relation Increased OJ ≥ 6 mm Increased OB ≥ 5 mm	Class II GHD 22.2% Non-GHD 25.9% GHD + non-GHD 29% Class II 71% Class I OJ 14% OB 5%	26.7% Class II OJ < 2 mm OB < 3 mm	None
Hodge et al. (2015) [42]	*Malocclusion* Angle’s Class I, II, III classification Increased OJ >2 mm Increased OB > 3 mm	31% Class II 6% Class III OJ 37% OB 37%	Class II 35% 6–11-year-olds 32% 12–17-year-olds	None
Kjellberg et al. (2000) [28]	*Dental Maturity* Measured as tooth formation, Demirjian method	*Dental maturity* GHD 11.1 ± 1.7 years Non-GHD 10.2 ± 1.8 years *Difference Dental age/Birth age* GHD Difference −1.0 Non-GHD Difference −1.3 GHD + non-GHD −1.2 years	No detailed data, A Finnish sample of 12-year-olds	Statistical significant differences in birth age vs. dental age between patients and control group (*p* < 0.001)
Partyka et al. (2018) [41]	*Dental Maturity* Matiegka and Lukasova method	*Dental maturity* The group starting treatment 138.97 ± 27.76 months The group in the course of treatment 153.23 ± 25.72 months *Difference Dental age/Birth age* All patients −9.70 ± 16.37 months The group starting treatment −18.82 ± 18.28 months The group in the course of treatment −2.70 ± 10.40 months	*Dental maturity* 141 ± 40 months *Difference Dental age/Birth age* + 3.98 ± 11.06 months	Dental age (maturity) of GHD patients is significantly delayed (*p* = 0.000) Statistical significant differences between birth age and dental age in patients starting treatment (*p* = 0.005)

**Table 4:** Anthropometric craniofacial characteristics—data abstracted from studies included in systematic review.

Reference	Methods of Study	Linear Measurements GHD Patients	Angular Measurements	Conclusions
Kjellberg et al. (2000) [28]	Lateral cephalograms, Bjork method 10 Linear and 12 angular measurements, 2 ratios.	s-n (mm) s-ba (mm) n-sp’ (mm) sp-pm (mm) sp’-gn (mm) tgo-ar (mm) gn-tgo (mm) ar-gn (mm) n-gn (mm) tgo’-tgo (mm)	Cranial n-s-ba n-s-ar Facial upper/lower s-n-ss NL/NSL s-n-sm s-n-pg ML/NSL ML/NL gn-tgo-ar ss-n-sm s-ar-tgo n-ss-pg	No significant differences were detected between the 28 non-GHD and 20 GHD patients. All linear measurements, except s-n and gn-tgo, were significantly smaller in the study group. A flat medial and lateral cranial base angle (*p* = 0.002) and large gonion angle (*p* = 0.002) were significant characteristics of studied patients. Both the mandible (*p* < 0.000) and maxilla (*p* = 0.004) were significantly retropositioned. The mandible showed an increase in the vertical inclination (*p* < 0.000).
Segal et al. (2004) [46]	Triangulation methods developed by Bookstein, 22 landmark points			The vertical proportions of untreated patients were significantly smaller in comparison with normal relatives *p* < 0.001; Deficit in facial proportions localized in the lower face. The vertical proportions of treated patients were not significantly smaller.
Funatsu et al. (2006) [2]	Two cephalometric radiographs- in centric occlusion and wide opening lateral; 12 lendmarks; 8 Linear and 5 angular measurements.	N-S, mm N-Me, mm N-ANS, mm ANS-Me, mm A-Ptm, mm Gn-Cd, mm Pog-Go, mm Cd-Go, mm	∠SNA, ∠SNB, ∠ANB, Mandibular plane to SN, Gonial angle	Ans-Me, <Gn-Cd,A’-Ptm’,Pog’-Go, Cd-Go were significantly smaller in boys and ANS-Me,Gn-Cd, Pog’-Go in girls in untreated group. Cg-Go was significantly larger; SNA, gonial angle were significantly smaller in boys; gonial angle in girls in short-term therapy. N-Me’, ANS-Me, A’-Ptm’, Pog’-Go, Cd-Go, gonial angle were significantly smaller in boys and ANS-Me, Cd-Go, gonial angle were significantly smaller but A’-Ptm’ was significantly larger in girls in the long-term. There was a significant difference between the untreated and long-term therapy in upper facial height, maxillary length and ramus high-scores increased with the duration of GH therapy
Choi et al. (2017) [45]	Lateral cephalograms at T0 before the treatment, T1 2 years after treatment, Pancherz’s method; 9 linear and 7 angular measurements	N-S S-Ba ANS-PNS Ar-Go Go-Gn Ar-Gn ANS- Me N-Me S-Go	Cranial base angle (N-S-Ar) Ramal angle (SN-ArGo) Gonial angle (Ar-Go-Me) SN-Go-Me SNA, SNB, ANB	Before treatment, boys had shorter N-S, *p* = 0.002; S-Ba, *p* = 0.004; Ar-Go, *p* = 0.012; Go-Gn, *p* = 0.008 and greater ANB, *p* = 0.018. Girls had shorter N-S, *p* = 0.001; Ar-Go, *p* = 0.010. Boys with GHD before treatment had skeletal Class II tendency. After treatment the sagittal skeletal relationship improved significantly in boys with GHD and ISS
Preda et al. (2019) [44]	Lateral cephalograms, 11 linear and 6 angular measurements	n-s s-ba n-ba n-sp pm-sp sp-gn gn-go ss–pm ss-ba s–pm pm-ba	SNA, SNB, ANB, ML–NL s–n–sm s–n–ss	SNA, SNB were significantly smaller (*p* < 0.001), ANB was higher (*p* < 0.001). S-n-ss and s-n-sm were significantly lower (*p* < 0.001). Linear measurements -N-s (*p* = 0047), sp-gn (*p* = 0.008), gn-go (*p* = 0.003), s-ba (*p* < 0.001), n-ba (*p* < 0.001) were significantly reduced.
Kim et al. (2021) [43]	Lateral cephalograms, 12 landmarks, 12 linear and 7 angular measurements	N-S S-BA N-BA N-ANS S-PNS ANS-Me N-Me S-go ANS-PNS Art-Go Go-Pog Art-Pog	N-S-Art. Art.-Go-Me N-S-Go-Gn SNA, SNB, ANB, S-N-Art-Go	Significant differences were at anterior, posterior, total cranial base length (N-S, s-ba, n-ba); upper posterior and posterior total facial heigh (S-PNS, S-go), mandibular ramus height and mandibular corpus length (Art-Go,Go-Pog) (*p* < 0.05). Significant differences were at saddle angle, gonial angle, mandibular plane angle, position of maxilla, SNB and ANB (*p* < 0.05).

**Table 5:** Quality assessment of studies using the Newcastle–Ottawa Scale (✯—star (point) awarded in the quality assessment).

Reference	Problem	Selection of the Study Groups				Comparability	Outcomes		Appraisal Score	Quality Category (Risk of Bias)
		Representativeness of Exposed Cohort	Sample Size Calculation	Non-Respondents	Ascertainment of the Factor		Assessment of Outcomes	Statistical Tests		
Kjellberg et al. (2000) [28]	malocclusion	✯	-	-	✯✯	✯	✯✯	-	6/10	Fair (medium)
	dental maturity	✯	-	-	✯✯	✯	✯✯	✯	7/10	Good (low)
	craniofacial characteristics	✯	-	-	✯✯	✯✯	✯✯	✯	8/10	Good (low)
Segal et al. (2004) [46]	craniofacial characteristics	✯	-	✯	✯✯	✯	✯✯	✯	8/10	Good (low)
Funatsu et al. (2006) [2]	craniofacial characteristics	✯	-	-	✯✯	✯✯	✯✯	✯	8/10	Good (low)
Hodge et al. (2015) [42]	malocclusion	✯	-	-	✯	-	✯✯	✯	5/10	Fair (medium)
Choi et al. (2017) [45]	craniofacial characteristics	✯	-	✯	✯✯	✯	✯✯	✯	8/10	Good (low)
Wójcik et al. (2018) [39]	caries	✯	-	-	✯✯	-	✯✯	✯	6/10	Fair (medium)
Wójcik et al. (2019) [40]	caries	✯	✯		✯✯	-	✯✯	✯	7/10	Good (low)
Partyka et al. (2018) [41]	dental maturity	✯	-	-	✯✯	✯	✯✯	✯	7/10	Good (low)
Preda et al. (2019) [44]	craniofacial characteristics	✯	-	-	✯✯	-	✯✯	✯	6/10	Fair (medium)
Kim et al. (2020) [43]	craniofacial characteristics	✯	-	-	✯✯	✯	✯✯	✯	7/10	Good (low)

## Data Availability

Not applicable.
